# Why Is There a Lack of Consensus on Molecular Subgroups of Glioblastoma? Understanding the Nature of Biological and Statistical Variability in Glioblastoma Expression Data

**DOI:** 10.1371/journal.pone.0020826

**Published:** 2011-07-28

**Authors:** Nicholas F. Marko, John Quackenbush, Robert J. Weil

**Affiliations:** 1 Department of Neurosurgery and Brain Tumor and Neuro-Oncology Center, Cleveland Clinic, Cleveland, Ohio, United States of America; 2 Department of Biostatistics and Computational Biology and Department of Cancer Biology, The Dana Farber Cancer Institute, Boston, Massachusetts, United States of America; Dana-Farber Cancer Institute, United States of America

## Abstract

**Introduction:**

Gene expression patterns characterizing clinically-relevant molecular subgroups of glioblastoma are difficult to reproduce. We suspect a combination of biological and analytic factors confounds interpretation of glioblastoma expression data. We seek to clarify the nature and relative contributions of these factors, to focus additional investigations, and to improve the accuracy and consistency of translational glioblastoma analyses.

**Methods:**

We analyzed gene expression and clinical data for 340 glioblastomas in The Cancer Genome Atlas (TCGA). We developed a logic model to analyze potential sources of biological, technical, and analytic variability and used standard linear classifiers and linear dimensional reduction algorithms to investigate the nature and relative contributions of each factor.

**Results:**

Commonly-described sources of classification error, including individual sample characteristics, batch effects, and analytic and technical noise make measurable but proportionally minor contributions to inconsistent molecular classification. Our analysis suggests that three, previously underappreciated factors may account for a larger fraction of classification errors: inherent non-linear/non-orthogonal relationships among the genes used in conjunction with classification algorithms that assume linearity; skewed data distributions assumed to be Gaussian; and biologic variability (noise) among tumors, of which we propose three types.

**Conclusions:**

Our analysis of the TCGA data demonstrates a contributory role for technical factors in molecular classification inconsistencies in glioblastoma but also suggests that biological variability, abnormal data distribution, and non-linear relationships among genes may be responsible for a proportionally larger component of classification error. These findings may have important implications for both glioblastoma research and for translational application of other large-volume biological databases.

## Introduction

Glioblastoma (GBM) is the most common primary malignant brain tumor in adults, and optimal surgical and medical management of this disease result in a mean survival of only 12–14 months [1], [2], [3], [4], [5]. Intense efforts over the past several decades to advance GBM therapy have resulted in only modest improvements in survival for patients with theses tumors, and the current management strategy remains attempted gross total surgical resection followed by radiation and adjuvant chemotherapy [6]. While the prognosis remains poor for most GBM patients, a small subset (10–25%) survive two or more years from the time of initial diagnosis [5], [6], [7], [8]. This variable response to standardized management suggests the existence of two or more major clinical subgroups of GBM patients with unique survival and response-to-therapy phenotypes. These subgroups are not readily identified by the current, histological grading and World Health Organization (WHO) classification schemes, prompting a search for alternate strategies for glioma classification.

The recent development of high-throughput molecular techniques for comprehensive characterization of tumor genomes and transcriptomes has been embraced by the translational neuro-oncology community and has been applied to the challenge of molecular GBM subclassification. Numerous investigators have reported successful identification of gene expression patterns characteristic of distinct tumor genomic profiles associated with unique clinical phenotypes [8], [9], [10], [11], [12], [13], [14], [15]. These results suggest that molecular analyses may improve prognostication in patients with GBMs and, more importantly, may identify subsets of GBM patients prospectively with distinct survival or response-to-therapy phenotypes.

Initial optimism that molecular classification tools represent a major breakthrough in GBM management has, more recently, been tempered by the lack of consistency and reproducibility of genomic signatures with putative associations to survival phenotypes. While multiple groups have reported the ability to predict patient survival accurately based upon specific gene expression signatures [8], [12], [13], [15], there is little overlap between the specific signatures reported by each group. Although it may be appealing to conclude that differences in the complex, multistep algorithms for gene selection can explain the variability among the specific genes comprising each reported molecular “survival fingerprint,” it is difficult to verify that analytic (rather than biologic or technical) differences are the principal determinants of this variability. Moreover, while hypothesis generation abounds regarding the potential biological significance of the genes in each profile, evidence supporting such hypotheses has heretofore been lacking. This problem is compounded by persistent uncertainty regarding the relative strengths and weaknesses of individual analytic models to capture phenotypically-relevant biology, and attempts to optimize these models is hindered by our incomplete understanding of cancer systems biology. Together, these observations can cast suspicion upon the biologic significance of the GBM expression signatures described by each group, and, consequently, upon the ultimate potential for clinical utility of this approach to molecular subclassification. While most translational neuro-oncologists believe that the future of GBM research lies in a better understanding of the molecular biology of these tumors, few agree on the specific genes of interest, the optimal approach to using genomic data to generate knowledge regarding the systems biology of these tumors, or the ideal strategies to apply this information to the classification and clinical management of GBM patients.

One step to address the challenges associated with analyzing and interpreting this data has been the development of a central repository of genomic and epigenetic data for GBMs. The Cancer Genome Atlas (TCGA) project [16] was designed as such a data repository, and GBM was the first tumor type to be cataloged and shared through the TCGA infrastructure [9], [14]. Public availability of this data increases both the number of investigators searching for novel genome/phenotype correlations in GBMs and the scope of biological hypotheses generated regarding such correlations; however, the accuracy, reproducibility, and utility of these investigations will remain uncertain until the factors responsible for the observed inconsistencies in GBM molecular classification can be identified, modeled, and prospectively addressed.

**Figure 1:**
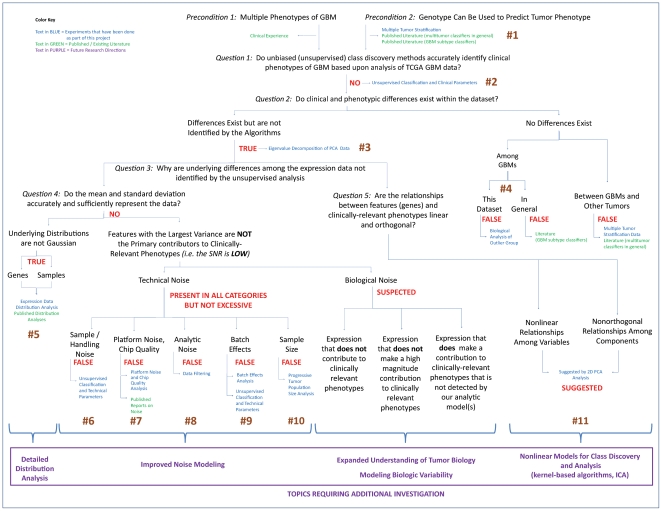
Logic Model for Analyzing Variability in the TCGA GBM Dataset. Annotated logic model, as applied in this investigation to the TCGA GBM data. The five fundamental questions forming the branch points of the logic model (see Discussion) are indicated in italics, and the answers to these questions are presented in red. Numbers (brown) corresponding to analyses 1–11 (see Results) are indicated at the locations where their results provide evidence. Analyses performed as a part of this investigation are indicated in blue, while supporting evidence from the literature is in green. Future directions for research are indicated in purple and are indicated below the corresponding branch of the model.

Comprehensive investigation of the potential sources of molecular classification errors in GBM requires questioning the fundamental assumptions regarding both the nature of the expression data itself and the analytic strategies used for its analysis. We hypothesize that a combination of biological and mathematical factors confound interpretation of this data, and we have undertaken a comprehensive investigation into the nature and relative contributions of these factors to inconsistencies in molecular classification of GBMs. To investigate our hypothesis, we constructed a logic model (Figure 1) to organize the major potential sources of error associated with current analyses of GBM expression data.

Logic models are narrative or graphical depictions of processes that communicate the underlying assumptions upon which an activity is expected to lead to a specific result. Such a model describes the logical linkages between elements of a process as a linear sequence of inputs, activities (in our case analytical steps), outputs, and outcomes [17], [18]. Once a program has been described in terms of a logic model, critical measures of performance can be identified. Having constructed our logic model, we then used as the basis for asking five (5) questions regarding the classification variability associated with the possible factors introducing error into any analysis of genomic data. Next, using publically-available (TCGA) GBM expression data [16], we designed and conducted eleven (11) mathematical analyses intended to address these five questions. We believe that this approach, which represents the first application of this type of analytic model to a large, publically-available gene expression database, provides a comprehensive framework for investigating and understanding the type, nature, and complex interrelationship among potential sources of error that may confound current strategies for molecular analysis and classification of GBMs.

## Results

Eleven analyses were conducted to test the logic model outlined in Figure 1. The design and results of these analyses are described below, and their positions in the logic model are indicated numerically on the figure. For reference purposes, an unannotated version of this figure has been included in the supplemental material (Figure S1).

### Multiple Tumor Stratification (#1)

**Figure 2:**
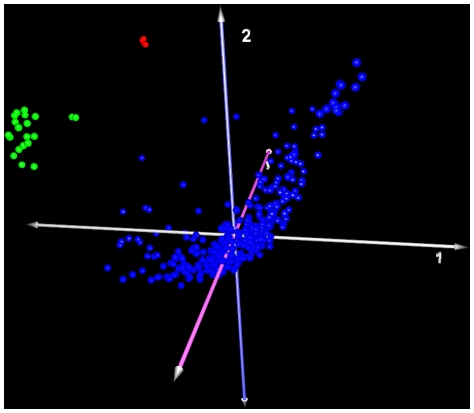
Multiple Tumor Stratification. PCA successfully separates glioblastoma (GBM, Blue) from renal cell carcinoma (RCC, green) and hepatocellular carcinoma (HCC, red) based upon expression profile.

Unsupervised classification using hierarchical clustering (HCL) [19] and principal components analysis (PCA) [20], [21] were performed on an Robust Multichip Analysis (RMA) –normalized [22], [23], [24] dataset containing 364 samples (340 GBM, 20 renal cell carcinoma [RCC], 4 hepatocellular carcinoma [HCC]). Both methods yielded excellent separation of the three, distinct tumor types based upon gene expression profiles (Figure 2). Next, minimally-supervised clustering using k-means clustering (KMS) [25] was performed. The value of k was set as 2 for this analysis, testing the ability to separate the GBMs from the aggregate group of RCC+HCC (i.e. GBM versus non-GBM) in a retrospective analysis. The analysis was repeated 100 times to minimize random classification effects that may be attributable to disproportionate allocation of tumor types in the population. This method achieved separation of the two groups with 99.6% accuracy (2/340 misclassifications, data not shown). Finally, the k-nearest neighbor clustering algorithm (KNNC) [26] was used to test the accuracy of prospective classification of novel samples as either GBM or non-GBM (k = 2) based upon expression profile. The KNNC was first trained with a randomly-selected set of 170 GBMs and 13 non-GBMs and was then tested using a novel set of 170 GBMs and 13 non-GBMs. This classifier achieved a 98.9% accuracy rate (2/170 misclassifications, data not shown). Together these results demonstrate the presence of a unique gene expression signature present within the TCGA data that is characteristic of GBMs and that is distinct from the molecular signatures of other malignancies.

### Unsupervised Classification and Clinical Parameters (#2)

**Figure 3:**
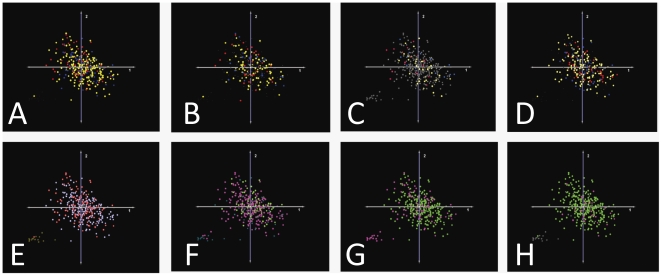
Unsupervised Classification (PCA) Colored by Clinical Phenotypes. GBMs do not segregate on PCA along any of 8 clinically-relevant parameters recorded in the TCGA database, which suggests that genomic signatures associated with these phenotypic differences are not identified accurately by this unsupervised, linear data reduction algorithm. A: Survival (Red = top 20^th^ percentile; Blue = bottom 20^th^ percentile; Yellow = intermediate percentiles). B: Time to Progression (Red = top 20^th^ percentile; Blue = bottom 20^th^ percentile; Yellow = intermediate percentiles). C: Time to Recurrence (Red = top 20^th^ percentile; Blue = bottom 20^th^ percentile; Yellow = intermediate percentiles). D: Karnofsky Performance Score (KPS) at diagnosis (Red = 100; Yellow = 80–90; Blue = 70 or less). E: Sex (Blue = male, Pink = female). F: Histologic Evidence of both Endothelial Proliferation and Necrosis (If yes, coded Green; if no, coded Pink). G: Adjuvant chemotherapy administered (Green = yes, Pink = no). H: Adjuvant radiotherapy administered (Green = yes, Pink = no). Note1: adjuvant therapy categories are not themselves phenotypic characteristics, but the need for these therapies may be a surrogate marker for underlying tumor biology and they are therefore included in this analysis. Note 2: for all images, spheres colored gray or brown = data not available.

**Figure 4:**
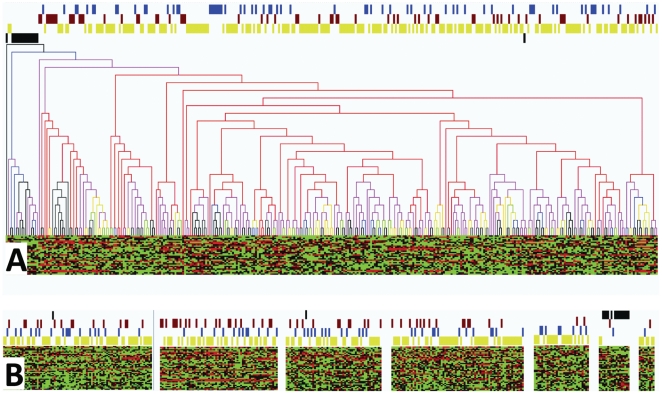
Unsupervised Classification (HCS, KMS) Colored by Survival Phenotype. The topmost color gradient bar displays the scale along which the Log_2_ (tumor/control) values for each gene are colorized in the dendrogram. The color bars immediately above the dendrogram branches indicate the survival phenotype group to which each sample belongs: Red = top 20^th^ percentile of survival time; Blue = bottom 20^th^ percentile; Yellow = intermediate percentiles, Black = survival not recorded. A: Hierarchical clustering with support (bootstrapping with 20 iterations) demonstrates no separation by survival phenotype. Support values for each node of the dendrogram also reveal significant inconsistency in clustering, suggesting underlying noise (black = 100% support, gray = 90–99%, blue = 80–89%, green = 70–79%, yellow = 50–69%, pink/red <50%). Only the topmost portion of the full heatmap is depicted in this figure. B: Clusters resulting from k-means support (k = 2, 10 iterations), again showing no segregation by survival phenotype.

Unsupervised classification using hierarchical clustering with bootstrapping support (HCS), KMS, and PCA was performed on the dataset containing 340 GBMs normalized to controls to investigate whether unbiased clustering algorithms would separate the population of GBMs into genomic subsets with corresponding, clinically-relevant phenotypes. Eight versions of each analysis were performed, each with samples colorized according to one of the eight clinical phenotypes with known or potential clinical significance recorded in the TCGA dataset. The results of the complete PCA analysis are presented in Figure 3, and representative results from the HCS and KMS analyses are given in Figure 4. These analyses demonstrate that three different unbiased class discrimination algorithms fail to segregate the GBM population into genomic subgroups correlated with any of the eight, clinically-relevant phenotypes for which clinical data is available.

### Eigenvalue Analysis (#3)

**Figure 5:**
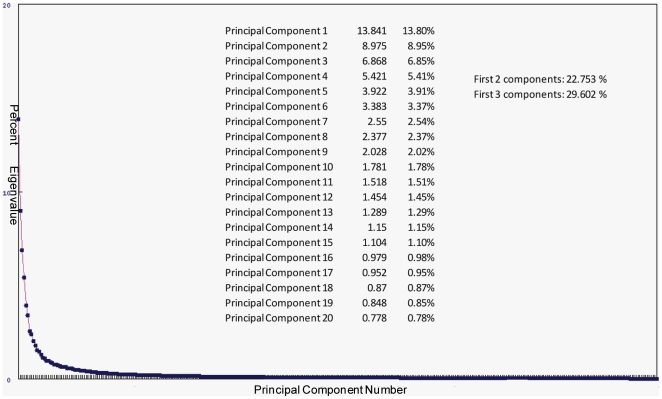
Eigenvalue Decomposition Analysis of PCA. Eigenvalues for all principal components are plotted, and numeric values for the eigenvalue percentages and cumulative percentages of the first 1–3 principal components are given. See text for discussion.

To study the extent to which the principal components (PC) describe the variability in the GBM population, we examined the eigenvalues resulting from the PCA matrix decomposition of the dataset containing 340 GBMs normalized to controls. Eigenvalues for each PC were computed both as a raw value and as a percent of the cumulative total eignevalue. The eigenvalue plot, as well as the individual eigenvalues and the eigenvalue percents associated with each of the first 20 PCs, are presented in Figure 5. This analysis demonstrates that the first three principal components, which serve as the basis for class discrimination represented by the 3-D PCA plot, account for only 29.6% of the total molecular variability of the GBMs. This suggests that this classification model does not capture or represent 70.4% of the genomic variability in this dataset. Moreover, it indicates that the most variable genes in this dataset, which theoretically contribute significantly to the first three PCs, may not have direct correlations with clinically-relevant phenotypes.

### Biological Analysis of the Outlier Group (#4)

**Figure 6:**
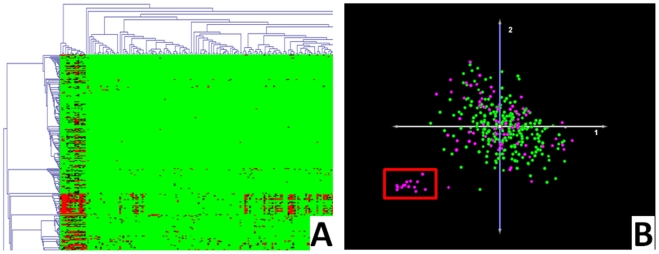
Outlier Group. A group of 14 outliers (red box) is consistently identified with various unsupervised clustering algorithms, including hierarchical clustering (A) and PCA (B). Note: a reproduction of Figure 3G has been used here and is colorized in an identical fashion.

HCS and PCA of the 340 GBMs (normalized to controls) demonstrated a cluster of 14 tumor “outliers” that consistently segregated from the remainder of the group in multiple analyses (Figure 6). We hypothesized that if there was a biological basis to this segregation, then more genes should be differentially-expressed between the outlier cluster and the remainder of the GBM population than would be expected by random chance. More importantly, functional annotation of the genes that are differentially expressed between the two groups should reveal enrichment of genes and categories believed to be associated with discrete GBM phenotypes. This analysis is qualitative in nature and limited in its power to draw definitive conclusions, but it represents one approach to investigate the hypothesis that valid, biological differences are present within the dataset and are contributing to the unsupervised tumor stratification.

The significance analysis for microarrays (SAM) [27], [28] algorithm with false discovery rate (FDR) = 0 was used to identify 2,501 over-expressed genes (Table S1A) and 2,704 under-expressed genes (Table S1B) in the outlier group relative to the remainder of the GBM population, more than would be expected by chance alone [2,501+2,704 = 5,205 genes identified by SAM vs. 172 (*p* = 0.01) or 857 (*p* = 0.05) that would be differentially regulated by pure chance]. EASE [29] overrepresentation analysis of the structural and functional annotations of these genes, including annotations from the Gene Ontology [30], [31] project, the Kyoto Encyclopedia of Genes and Genomes [32], [33], and GenMAPP [34], [35], demonstrated statistically-significant (EASE score <0.05) overrepresentation of 473 categories, many of which have plausible roles in differential tumor phenotypes (Tables S1C and S1D). Comparison of this gene list and annotation profile with a profile that our group previously identified as differentially-expressed between GBM survival phenotypes [8] demonstrated overlap in 8 (15%) genes and 6 (46%) categorical annotations (Table S1E). Together, these results provide qualitative evidence to suggest that genomic differences with potential phenotypic significance do exist among the TCGA GBM data and that at least some of this biological information influences the results of the clustering algorithms.

### Expression Data Distribution Analysis (#5)

One assumption common to most linear classifiers used in microarray analysis is that the values of the variables upon which classification is based conform to a Gaussian distribution [21], [36]. To test this assumption, we examined the distributions associated with the RMA normalized, Log_2_ (tumor/normal) data. We studied the distribution characteristics of the variables (gene expression values) in the dataset for the complete expression matrix (17,172 genes×340 samples = 5,838,480 variable values) as well on a per-gene and per-sample basis. We characterized the distribution by computing standard descriptive statistics as well as skew and kurtosis measurements (and descriptive statistics for each of these measurements). The results of this analysis are summarized in Table 1.

**Table 1:** Descriptive Statistics for the TCGA GBM Distribution.

T	RMA Normalized, Log2 (Tumor/Normal)			Standard Gaussian (normal)
	By Gene	By Sample	Overall	
**Distribution**				
Mean	0.03	0.03	0.03	0.00
Median	0.00	0.04	0.03	0.00
SD	0.45	0.91	0.92	1.00
**Skew**			−0.21	0.00
Mean	0.64	−0.18		
Median	0.50	−0.20		
SD	1.14	0.33		
Min	−7.57	−1.00		
Max	14.13	1.06		
**Kurtosis**			6.45	3.00
Mean	3.07	5.82		
Median	0.86	5.88		
SD	9.97	1.14		
Min	−1.60	2.69		
Max	236.58	9.35		

**Figure 7:**
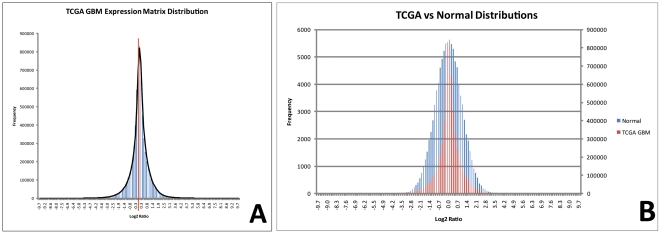
Distribution of the Complete Gene Expression Matrix for the TCGA GBM Data. A: TCGA GBM expression matrix histogram. The zero position on the abscissa is indicated with a red meridian. Careful inspection (particularly at the apex) reveals the negative skew. B: Overlay of the TCGA GBM distribution and the standard Gaussian (normal) distribution. This representation, in particular, demonstrates the leptokurtosis of the TCGA GBM data distribution. Scales of the ordinates have been adjusted to facilitate distribution overlay.

Note that both the mean and median are approximately equal to zero and the standard deviation is approximately equal to 1. Measurements of skew and kurtosis deviate from those expected for a Gaussian distribution, and test statistics indicate that these deviations are statistically significant (test statistic >2 approximately corresponds to *p*<0.05, see text). These results are depicted graphically in Figure 7.

**Figure 8:**
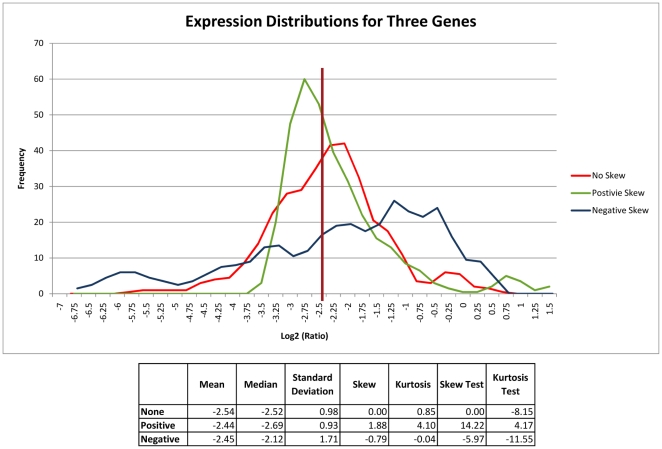
Distributions of Three Sample Genes from the TCGA GBM data. The expression distributions across all 340 GBM samples of three, representative genes with approximately equal means, medians, and standard deviations are plotted. Note that, for each sample, the mean and median are nearly equal and the standard deviation is approximately equal to 1. These descriptive statistics suggest a Gaussian distribution. However, the three distributions are significantly unequal, a finding only reflected in the measurements of skew. Mild kurtotic effects can also be observed. Test statistics for skew and kurtosis are calculated as described in the text. (Note: The negatively skewed sample gene is TCEAL2]211276_at], the positively skewed sample gene is CENTA1 [90265_at], and the unskewed gene is CYFIP2 [21578_s_at].).

The distribution analysis demonstrates that the TCGA GBM data deviates from a pure Gaussian distribution both in the overall expression matrix (Figure 7) and in the per-gene (Figure 8) and per-sample (not shown) analyses. Examination of the RMA-normalized, log_2_(ratio) data reveals that the complete expression matrix is centered around a mean and median of 0.03 with a standard deviation of 0.92, suggesting a normal (standard Gaussian) distribution. However, the skew of this distribution is −0.21, an effect that is easily overlooked on visual inspection (Figure 7A). While this value may not initially appear to be of significant magnitude, the statistical significance of the degree of skew must be considered in the context of the number of variables (n) associated with the distribution. A test statistic, reflecting the number of standard errors of skew (SES) [37] away from zero, can be computed from equation 1:
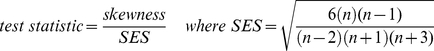



This equation essentially performs a two-tailed test of skew≠0, where a test statistic value of ±2 corresponds to *p* = 0.05. Using *n* = 5,838,480 (the total number of values *comprising* the expression matrix), *SES* = 0.001 and the *test statistic* = −207.2, indicating that the distribution has a statistically-significant degree of skew. Similarly, the kurtosis of the distribution is calculated at 6.45 (excess kurtosis = 3.45). The statistical significance of the magnitude of the kurtosis must also be considered in the context of the number of variables (*n*) associated with the distribution, and a test statistic reflecting the number of standard errors of kurtosis (SEK) [37] away from zero, can be computed from equation 2:
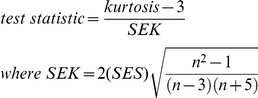



For *n* = 5,838,480, *SEK* = 0.002 and the *test statistic* = 1701.6, indicating that the distribution has a highly statistically-significant degree of leptokurtosis (Figure 7B).

When the skew and kurtosis as well as their corresponding test statistics are computed for each gene (a per-gene analysis, where n = 340), the mean skew is 0.64 (mean test statistic = 4.84) and the mean kurtosis is 3.07 (mean excess kurtosis 0.07, mean test statistic = 0.27). A similar analysis done on a per-sample basis (n = 17,172) demonstrates a mean skew of −0.18 (mean test statistic = −9.64) and a mean kurtosis of 5.82 (mean excess kurtosis 2.82, mean test statistic = 75.34), suggesting significant deviations from a pure Gaussian distribution in both analyses. Examples of the diversity of skew and kurtosis of gene distributions are given in Figure 8. While the classification implications of these deviations from the Gaussian distribution require further investigation [36], [38], it is apparent from this analysis that the TCGA GBM data violates the Gaussian assumption that is a fundamental precondition of most linear classifiers.

### Unsupervised Classification and Technical Parameters (#6)

**Figure 9:**
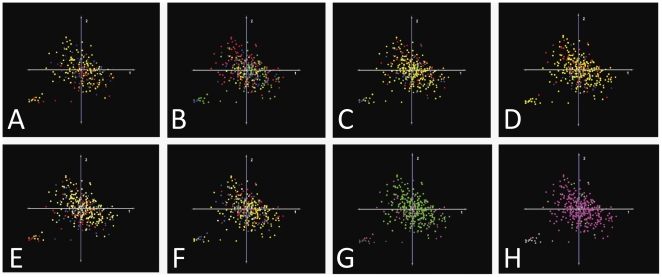
Unsupervised Classification (PCA) Colored by Technical Phenotypes. GBMs do not segregate on PCA along any of 8 technical parameters recorded in the TCGA database. This suggests that these factors are not primary contributors to erroneous misclassification. A: Tissue Sample Size (Red = top 20^th^ percentile; Blue = bottom 20^th^ percentile; Yellow = intermediate percentiles). B: Clinical Center of Origin (By TCGA ID number: Red = 02, Green = 06, Blue = 08, Yellow = 12, Purple = Other). C: RNA Concentration (Red = top 20^th^ percentile; Blue = bottom 20^th^ percentile; Yellow = intermediate percentiles). D: Optical Density 260/280 nm (Red = top 20^th^ percentile; Blue = bottom 20^th^ percentile; Yellow = intermediate percentiles). E: Ribosomal 28S/18S (Red = top 20^th^ percentile; Blue = bottom 20^th^ percentile; Yellow = intermediate percentiles). F: RNA Index Number (Red = top 20^th^ percentile; Blue = bottom 20^th^ percentile; Yellow = intermediate percentiles). G: >50% Tumor Cells in Sample (Green = yes, Pink = no). H: Prior Treatment (Green = yes, Pink = no). Note: in all figures, gray = data not available.

Unsupervised classification using PCA was performed on the dataset containing 340 GBMs (normalized to controls) to investigate whether unbiased clustering algorithms would separate the population of GBMs into subsets corresponding to technical differences related to the samples or to their processing. Eight versions of the analysis were performed, with samples colorized according to one of eight technical parameters that could introduce classification error. The results of this analysis (Figure 9) demonstrate that these eight technical parameters do not appear to be major contributors to the first three principal components and are therefore unlikely to be primary contributors to classification errors or inconsistencies.

### Platform Noise and Chip Quality Analysis (#7)

Multiple technical variables associated with sample preparation, hybridization efficiency, physical array quality, and image acquisition can contribute to technical noise and subsequently to classification error. These effects have been studied extensively [39], [40], [41], [42], [43], [44], [45], [46], [47], [48], and numerous modeling and optimization strategies have been suggested to estimate and to minimize technical noise [40], [49], [50], [51], [52], [53]. Implementation and optimization of such modeling is beyond the scope of this investigation, but semi-quantitative estimation of the magnitude and effects of technical noise are useful to illustrate their relative importance for this dataset.

For each gene in each array, the MAS5 algorithm [54] calculates a discrimination score (R*_i_*) based upon the relative signal levels measured for the perfect match (PM) and mismatch (MM) cells for each probeset. A one-sided Wilcoxon's Signed Rank Test is used to calculate a p-value associated with R*_i_*. Comparison of the p-value with user-defined threshold values (α_1_ and α_2_) serves as the basis for the algorithm's present/absence call, and the p-value itself can be conceptualized as a measure of confidence that the measured signal is the result of valid hybridization rather than background noise. Qualitative assessment of the extent of the noise in the TCGA dataset was performed by calculating the percentage of genes in the expression matrix for which *p*>0.05 (41.76%) and *p*>0.01 (51.50%). This analysis suggests that approximately 40–50% of the individual expression measurements in the matrix are not significantly different from background. We performed similar analysis on smaller sets of previously-published expression data for GBMs (n = 20) [8] and for low-grade gliomas (n = 23) [55] and found similar percentages for each dataset (45.98%/57.35% and 50.43%/64.16%, respectively). Similarly, we calculated the percentages for genes called present, absent, and marginal by the MAS5 algorithm (with α_1_ = 0.05, α_2_ = 0.065, τ = 0.015) for the TCGA dataset and for the two comparison datasets and again found comparable values in each category (Table 2).

**Table 2:** Analysis of MAS5 *p*-values as Markers of Background Noise.

	TCGA GBMs	Comparison GBMs	Comparison Low Grade Gliomas
**Call = Present**	58.2%	44.5%	49.6%
**Call = Absent**	40.6%	54.0%	48.6%
**Call = Marginal**	1.2%	1.5%	1.9%
***p*** **>0.05**	41.8%	46.0%	50.4%
***p*** **>0.01**	51.5%	57.4%	64.2%

P-values were computed by the Affymetrix MAS5 algorithm using a one-sided Wilcoxon Signed Rank Test. “Call” refers to the Affymetrix Present/Absent call. Comparison GBMs were initially reported in Marko et al., 2008 [8], and comparison low-grade gliomas were reported in Marko et al. 2009 [55].

Next, we used the *p*-value associated with R*_i_* as the basis of a semi-quantitative analysis examining the effects of technical noise on clustering results. We recognize that this is not a comprehensive marker of all possible sources of technical noise, but it is a useful index of one dimension of this noise and has been used as a surrogate marker for array noise in other investigations [49]. We computed the mean of the *p*-value (*p*_m_) for each gene across the set of 340 arrays and subsequently filtered out genes for which *p*_m_>0.05 from the RMA-normalized dataset used in our other analyses. We then performed PCA on this filtered dataset and found no qualitative improvement in clustering into survival phenotypes (data not shown). The sum of the first three eigenvalue percentages from this analysis was slightly better than that of the unfiltered data (34.4% vs 29.6%). Together, the results of these analyses suggest that technical noise is present within the TCGA dataset but that it does not appear to be the major factor confounding clustering into clinically-relevant phenotypes.

**Figure 10:**
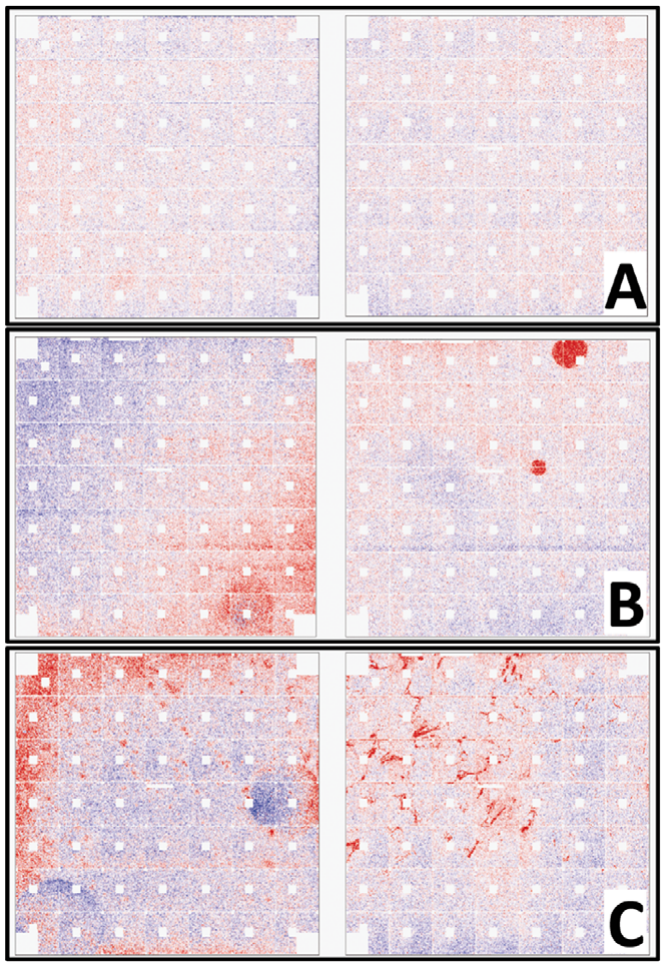
Assessment of Array Chip Quality. Pseudo-chip images created from the RMA probe-level modeling (PLM) residual values were used to screen chips for quality. The presence of either positive (red) or negative (blue) residuals indicates some degree of deviation from the ideal (zero residual) value and therefore suggests some degree of error in the measurement. Technical problems may result in nonrandom regions of unusually large residuals. Depicted are examples of chips considered to be of good (A), intermediate (B), or poor (C) quality in our analysis.

Chip quality was also performed to identify areas of chips with inhomogeneous signal characteristics. This can be attributable to physical errors on the chips, fluidics errors, regional binding inconsistencies, or other regional effects. We first examined the virtual chip images created using the RMA Express [22] software package's QA tools. These pseudoimages are derived from the RMA algorithm's residual PLM values and are capable of highlighting areas of inhomogeneous signal on the array chips. We considered chips to have significant regional error if inhomogeneities were identified on at least part of 5 or more of the 49 subgrids (∼10%) of the array chip (Figure 10). A total of 24 chips (7.1%) with such regional errors were then excluded from subsequent clustering analysis. PCA clustering demonstrated no apparent improvement in ability to segregate survival phenotypes after exclusion of these chips (data not shown) and no significant improvement in the sum of the eigenvalue percentages associated with the first three principal components (27.6% vs 29.6%). We then repeated this analysis using more stringent criteria for chip quality, including either evidence of regional errors in 3 of 49 (∼6%) subgrids or other patterns potentially suggestive of nonbiological inconsistency (see Figure 10). This resulted in exclusion of 108 (31.8%) arrays but did not improve qualitative classification (data not shown) or cumulative eigenvalue percentages (27.8% vs. 29.6%) in subsequent PCA analysis. Both analyses, however, did result in a change in morphology of the PCA plot (relative to the plot of the unfiltered data, data not shown), the significance of which requires further investigation. Together these analyses suggest that platform noise and chip quality are present in the TCGA GBM dataset but may not be the primary contributors to inaccuracies in sample classification.

### Data Filtering (#8)

**Figure 11:**
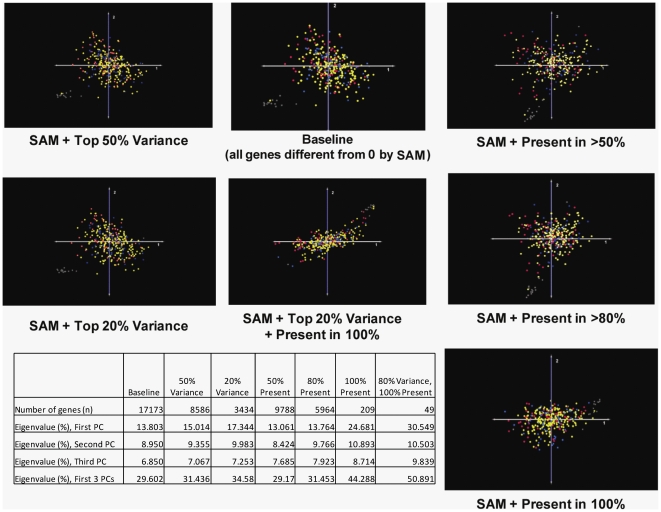
Data Filtering Analysis. Various combinations of data filtering strategies are applied to the TCGA GBM dataset (as indicated below the images) and PCA plots colored by survival phenotype (Red = top 20^th^ percentile; Blue = bottom 20^th^ percentile; Yellow = intermediate percentiles) are generated. A summary of the eigenvalue data is presented in tabular form. This analysis suggests that technical variability introduced by genes with relatively homogeneous or absent expression values is a source of variability but does not appear to be the primary factor impeding accurate phenotypic classification.

Two potential sources of statistical noise that may impede classification are genes with either relatively static expression levels throughout the data set or genes for which expression values are absent in a significant portion of the expression matrix. We performed a series of data filtering analyses designed to investigate the effects of these variables, in isolation and in combination, on unsupervised classification into survival groups. We first performed PCA using the baseline data set of 340 GBMs (relative to controls) and then repeated this analysis using progressively stringent variance and present/absent data filters on the normalized dataset. These filters removed the least variable 50% or 80% of genes (variance filter) or limited inclusion of genes to those with measurable signal present in >50%, >80%, or in 100% of samples (percent present filter). Data filtering trials were performed with individual filters or with combinations of the variance and percent present filter in place at various individual thresholds. PCA was performed on each of the filtered datasets, and the ensuing classifications were evaluated qualitatively by examining the plots of the first three principal components (colorized by survival) and semi-quantitatively by analyzing the eigenvalue percentages associated with each of the first three principal components in each of the trials (Figure 11). The results of the semi-quantitative analysis show a trend toward increasing eigenvalue percentage as progressively more stringent criteria are applied, suggesting that statistically “noisy” genes do contribute, to some degree, to the variability represented by the first three principal components. The qualitative analysis demonstrated that, while filtering changed the overall morphology and orientation of the sample cloud in 3-dimensional, PCA space, the observed reduction in variability did not improve the accuracy of stratification into clinically-relevant subgroups. Overall, these results suggest that technical variability introduced by genes with relatively homogeneous or absent expression are a source of classification variability, but these do not appear to be the primary factors impeding accurate phenotypic classification of GBMs in the TCGA data set.

### Batch Effects Analysis (#9)

The TCGA GBM data set includes 340 samples derived collected from patients treated at multiple clinical centers and processed at one of several sample preparation laboratories. Combining the data from such samples may introduce batch effects into the concatenated data set. This phenomenon has been well described by array statisticians [56] but is sometimes overlooked by clinician-scientists using large, public datasets for hypothesis generation and testing. We analyzed the potential contribution of batch effects in the TCGA GBM data to tumor misclassification using both a “bottom-up” and a “top-down” approach. For the “bottom-up” analysis, we first prepared several data subsets in which samples were matched for approximate tissue size, OD260/280, 28S/18S, and RNA integrity number (RIN). We held constant either clinical center of origin or TCGA batch number while varying the other parameter. PCA was performed on the data subsets, and samples were colorized by the variable parameter. This approach facilitates qualitative exploration of the independent batch effects associated with either clinical center of origin or TCGA batch number, although it is limited by the scope of the technical data reported in TCGA as to the number of technical parameters that can be matched or controlled. The results of these analyses demonstrate no gross clustering trends associated with either center of origin or with TCGA batch, although caution must be used in interpreting this data because the qualitative nature of the analysis limits the extent to which batch effects can be studied.

**Figure 12:**
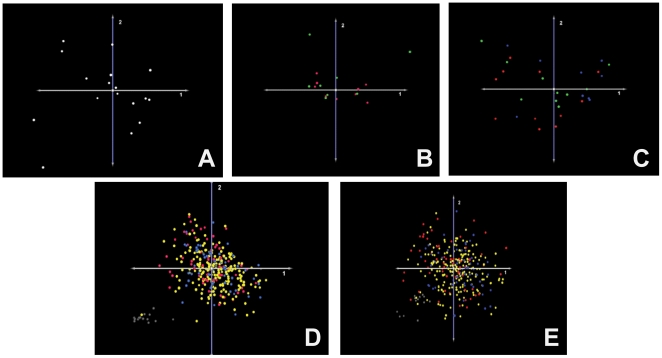
Batch Effects Analysis. A–C: Analysis of subsets (A = baseline subset) matched for all technical parameters except for either Hospital of Origin (B; by TCGA Hospital code, Green = 02, Red = 08) or TCGA Batch (C; Green = batch 1, Blue = batch 2, Red = Batch 5). Samples do not segregate by either technical variable, even when all other technical variables are controlled. This suggests that these variables are not major contributors to the first three principal components. D–E: The ComBAT algorithm was used to correct for batch effects. This changes the distribution along all 3 principal components (suggesting that batch effects may contribute to these components) but does not improve classification into survival phenotype, (suggesting that batch effects are not the principal factor confounding the PCA). Note: D–E are colorized by survival phenotype, as in Figure 3A.

Recognizing the limitations of the “bottom-up” analysis, we also performed qualitative and semi-quantitative analysis of batch effects using a “top-down” approach. We assumed the presence of some batch effects associated with clinical center of origin and TCGA batch, attempted to correct empirically for such errors, and compared the PCA plots and the eigenvalue data before and after correction to obtain a *post hoc* estimate of the classification impact of batch effects. We implemented this analysis by first performing baseline PCA analysis (colorized by survival) with eigenvalue decomposition on the complete set of 340 GBMs. Next, we applied the ComBAT algorithm [57], a tool that uses a Bayesian approach based upon empirical and parametric priors to reduce batch effects. We ran ComBAT using TCGA batch as the primary effect and clinical center of origin as the covariate. We repeated the PCA and eigenvalue analyses on the “corrected” dataset. The results of this analysis suggest that some center-of-origin and TCGA-batch-number batch effects are present in the concatenated data set. The result of correction for these effects can be observed qualitatively by comparing the pre- and post-combat PCA plots (Figure 12). The latter appears to demonstrate a reduction in correlation between the first two principal components, suggesting that batch effects may contribute to the identity and subsequent stratification of samples along these two components in some underlying and correlated fashion. Notwithstanding, the application of ComBAT does not result in qualitative improvement in phenotypically-significant (survival) clustering, suggesting that batch effects are not the primary contributor to classification error. This finding is verified by semi-quantitative analysis using the eignevalue data, which shows only a slight improvement in the sum of the first three eigenvalue percents (31.2% vs 29.6%).

### Progressive Tumor Population Size Analysis (#10)

**Figure 13:**
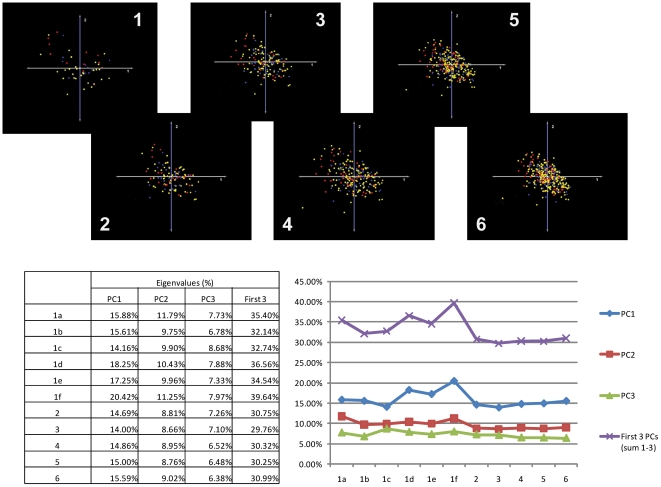
Progressive Tumor Population Size Analysis. PCA plots (colored by survival phenotype, Red = top 20^th^ percentile; Blue = bottom 20^th^ percentile; Yellow = intermediate percentiles) show no qualitative change in sample distribution as equal subsets are added. 1a–f refers to individual analyses of the six, equal subsets that were progressively added. Graphical representations of each of these subsets have been omitted in the interest of space and are, instead, illustrated by a single, representative, graphical description of subset 1a (labeled 1). From this point, the numbers correspond to the number of subsets present in the analysis. The eigenvalues associated with each subset and with the combined populations show no significant changes as additional samples are added (tabular, bottom left; graphical, bottom right). PC, principal component. This analysis demonstrates that the incorporation of additional samples causes some increase in noise, although this does not appear to be a major factor contributing to the first 3 principal components.

Although increasing the number of samples in a microarray experiment may increase classification power in some instances, this presupposes that the signal-to-noise ratio of the samples is higher than those of the base set. If the opposite is true or if the SNRs are approximately equal, then increasing population size may either disproportionately introduce noise that could impede classification or may have little to no added effect, respectively. To study the possibility of this effect in the TCGA GBM data, we used a random number generator to randomly divide the RMA-normalized, 340 tumor dataset into 6 subgroups of approximately equal size (n = 57×4, n = 56×2). We then progressively combined the subsets, creating five sub-populations of 114, 171, 228, 284, and 340 samples. Qualitative PCA and eigenvalue analysis were performed on each of the six subsets and on the five sub-populations. The results of these analyses demonstrate approximately stable magnitude of the eigenvalue percentages associated with each of the first three principal components in all subsets and concatenated sub-populations, and qualitative analysis of the PCA data does not demonstrate appreciable improvement (or decline) in phenotypic classification accuracy as the population size increases (Figure 13). These findings suggest that there is little relationship between tumor population size and classification accuracy for the TCGA GBM expression data.

### Two-Dimensional Analysis of Principal Components (#11)

A necessary condition for successful class discrimination by linear classifiers or data reduction algorithms is that the relationships between the variables used for classification must be linear [36]. Additionally, many data reduction algorithms require that the relationship between the most important variables (or the aggregate vectors that represent them) be orthogonal. PCA is among the most commonly-applied class discrimination and data decomposition algorithms used in GBM expression analysis, and it is an example of a linear classifier requiring that both conditions be satisfied [21]. Here, the former condition frames the data reduction as a change-of-basis problem, while the latter allows linear algebra techniques to be applied to the decomposition in orthonormal space. While these requirements limit the application of PCA to datasets whose variables comply with this underlying structure, they can also be exploited to investigate the relational structure among significant variables (or, at least, among those with the largest variance) in an expression data set by comparing the actual to the expected outcomes. We used PCA to investigate the validity of the assumptions of linearity and orthogonality for the TCGA GBM expression data set.

**Figure 14:**
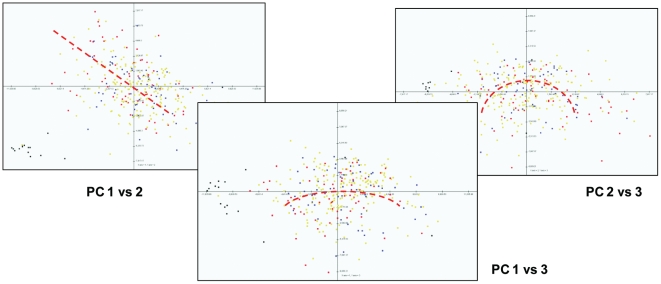
2-Dimensional Plots of Principal Components 1–3. Two-dimensional plots of the first three principal components of the TCGA GBM dataset (colored by survival phenotype, Red = top 20^th^ percentile; Blue = bottom 20^th^ percentile; Yellow = intermediate percentiles). These plots may suggest nonlinear and/or nonorthogonal relationships among the features (genes) used for classification. See text for detailed discussion.

When the underlying data relationships are both linear and orthogonal, two-dimensional plots of successive principal components (PC) should demonstrate that the samples display, on average, a linear relationship to one another with the regression line having a y-intercept and slope that approximate zero. Using the RMA-normalized data set of all 340 GBMs, we performed PCA and then examined the three, two-dimensional plots of the first three principal components (PCs 1 vs. 2; 1 vs. 3; and 2 vs. 3; Figure 14). Examination of the PC 1 vs. 2 plot suggests a linear correlation of the data but with a regressed slope <0. This effect can be observed when both PCs are influenced by one or more correlated variables and suggests that the first two principal components may not be purely orthogonal [21]. Examination of the PC 1 vs. 3 and PC 2 vs. 3 plots suggest qualitatively that the samples may be better represented by a nonlinear regression (i.e. cluster better around an arc than along a straight line). This artifact, sometimes referred to as a “horseshoe effect,” can be observed when the relationship between the variables contributing to the principal components is non-linear [21], [58], [59]. Although these observations require further investigation, examination of the 2-D PC plots raises the possibility of non-linear relationships between variables and/or non-orthogonal data structure within the TCGA GBM expression data that may explain some of the inconsistencies or failures of robust linear classifiers.

## Discussion

### The Role of Transcriptomic Data in Phenotype Prediction

Laboratory techniques for analyzing the transcriptome, including RT-PCR and microarray analysis, have evolved into some of the most robust and efficient means of collecting large quantities of molecular data from biological sepcimens. Because assays of the transcriptome are believed to provide a comprehensive window through which cellular physiology can be studied, a great deal of effort and resources have been devoted to large-scale transcriptomic analyses of human disease. It is essential to realize, however, that these investigations, while comprehensive, are unlikely to be exhaustive in their ability to predict phenotype. Post-translational processes also have phenotypic influence, and predictive models that include this data are likely to have improved accuracy when compared to transcriptome-only models. Notwithstanding, because genotype influences phenotype, the transcriptome is likely to contain valuable information on which predictive models can be built. The objective of this investigation is to better understand the potential sources of error in such models and to suggest strategies for reducing this error. Whether applied to transcriptomic data in isolation or to such data applied in a more comprehensive framework, an improved understanding of these error sources can only improve the predictive ability of molecular models that incorporate transcriptomic data.

### The Noise Problem and its Implications

Large scale molecular datasets, such as those generated by whole-genome expression analysis, have been heralded by the translational oncology community as an important step toward using tumor biology to guide disease diagnosis and therapy. Because genomic profiles and phenotype are related, it is logical that molecular signatures associated with specific phenotypes can be identified retrospectively and used to identify specific phenotypes with clinical relevance prospectively. Translational studies performed in the context of this paradigm have succeeded in both domains, retrospectively identifying differential patterns of gene expression between tumors from patients with distinct phenotypes and prospectively using such signatures to classify accurately novel tumor samples into appropriate phenotypic categories [8], [11], [12], [13], [14], [60], [61].

Initial enthusiasm inspired by these investigations has tempered, however, as repeated attempts at molecular subclassification of various malignancies fail to validate the putative role of genes identified as relevant by these classification schema and remain unable to reproduce consistent genomic signatures associated with similar clinical phenotypes. We believe that these two observations, the successful prospective predictive ability of gene expression data and the inconsistencies in the specific expression patterns on which such classifications are made, reflect two distinct and equally-important characteristics of large scale molecular datasets. The predictive power of classifiers based upon molecular signatures [62] demonstrates both the wealth of biological information contained in these datasets and the robustness of the analytic models applied to the problems of classification based upon this data. This finding validates the enthusiasm with which these investigations are undertaken and illuminates their potential utility as clinical tools. The inconsistencies, however, demonstrate the presence of one or more sources of noise within the dataset, where noise is defined as the presence of measured variable values (i.e. gene expression values) that do not contribute to the intended molecular classification. Despite thorough investigation, much remains unknown about the nature and magnitude of the noise, highlighted by the observed inconsistencies in identifying specific genes and gene expression signatures useful for classification. While the latter challenge is surmountable, it risks overshadowing the former strength unless the sources of such noise can be appropriately identified, modeled, and suppressed in future translational molecular oncology investigations. This problem spurred the present investigation.

### Data Quantity and Quality Relationships in Translational Molecular Biology

Strategies employed to reduce noise and improve the accuracy and precision of genome-based classifiers have focused on reducing error primarily by increasing the quantity of available data used in molecular classification investigations. Large, publically-available repositories of molecular data spanning a large number of pathologies were developed, including the Gene Expression Omnibus (GEO) [63], [64], [65] and The Cancer Genome Atlas (TCGA) [16]. This approach is consistent with traditional models of biological and clinical research, where improvements in power and reduction in error are achieved primarily through increases in sample size.

Large-scale, molecular data sets differ fundamentally from traditional biological and clinical data sets, however, in the relationship between the number of samples being studied (*n*) and the number of variables measured per sample (*p*). In traditional datasets, relatively few variables are tracked across a proportionately greater number of samples (*n>p*). The inverse is true of modern molecular datasets, where the number of variables measured per sample (typically expression levels of individual genes) far exceeds the number of samples in a given investigation [62] *(p≫n)*. This difference calls into question the validity of traditional models of error and noise reduction through increasing sample size, and two types of gross evidence appear to validate these concerns. First, statistical investigations regarding selection of sample size in microarray experiments, the details of which are beyond the scope of this discussion, suggest that large sample sizes may not always be necessary to draw meaningful biological conclusions [66], [67], [68], [69], [70], [71], [72]. Second, there is little evidence to suggest that classifiers derived from more recent investigations using large numbers of gene expression profiles [11] are more powerful or more robust than those generated from investigations performed using fewer samples [8]. Together, the combination of these theoretical, statistical, and practical observations suggest that increasing sample quantity may be neither necessary nor sufficient to reduce noise and error in analyses of large-scale molecular datasets.

If increasing sample quantity is not necessarily the primary method for reducing noise and error, then data quality must be improved. The concept of the “quality” of expression data is complicated by the fact that the experimental utility of a given dataset is relative to the biological hypothesis that it is being used to test. A molecular dataset that may be ideal to answer one question may contain too much noise to test an alternate hypothesis. In addition to stressing the importance of appropriate, initial study design in molecular analyses, this argument suggests that the concepts of error and noise have a dynamic component (that is relative to a given investigation) and that error modeling may need to be individualized [49] for a particular question addressed using a specific dataset. This reasoning may explain some of the theoretical underpinnings of the heterogeneity and variability noted in the core gene expression patterns employed by modern molecular classifiers.

Another possibility is that cancers, such as GBM, are much more molecularly diverse than we have previously recognized. While there may be some common molecular events contributing to the development and progression of the disease, it may be that the clinical phenotypes are mediated by large numbers of interacting genes and that this diversity would require even larger patient populations to identify the subtle signals that may be present. Alternately, it may be that a more hypothesis-driven, pathway-based approach to identifying phenotypically-relevant subclasses is necessary.

### Phenotypic Correlation as the Benchmark

Identification of genotypic tumor subgroups with phenotypic significance has been used as the benchmark for “successful” molecular tumor subclassification in this investigation. This strategy has been chosen because it reflects the real-world needs of translational neurobiologists and neuro-oncologists. These investigators, who attempt to identify molecular markers in glioblastoma patients that define discrete diagnostic, prognostic, or response-to-therapy subpopulations, represent a significant proportion of the target audience of the TCGA project and are currently among the most frequent consumers of the gene expression data housed in this database. While phenotypic correlations of genomic data are important in nearly all avenues of research conducted using such databases, we recognize that the benchmark we have chosen may not be ideally suited to the specialized needs of some mathematicians, basic scientists, and systems biologists who may be analyzing the TCGA data with non-clinical objectives. Notwithstanding, even investigators with such alternate aims may benefit from the conclusions drawn from this analysis.

### Logic Models and Error in Tumor Classification

Although specific sources of noise and their relative relationships may vary across diverse datasets and for specific clinical questions, there must be some logical framework through which to approach the identification of such confounding data. To address this challenge, we created a logic model we believe is useful for analyzing many of the potential sources of noise that result in inconsistent phenotypic classification of malignancies based upon gene expression data (Figure 1).

Our model is predicated upon two, necessary preconditions; that distinct phenotypic groups exist within the dataset and that genomic profiles can be used to predict phenotype. Assuming these conditions are met, we asked five basic questions, the answers to which frame a logic model for investigating the potential sources of noise that impedes reproducible classification into clinically-relevant phenotypes based upon molecular data. Finally, we apply this logic model to the TCGA glioblastoma expression dataset to investigate the potential sources of noise specific to this dataset and to the challenge of molecular classification of GBM into subgroups with distinct, clinically-relevant phenotypes. This exercise serves two purposes. First, it provides a specific analysis of the potential sources of noise and error that have been confounding attempts at reproducible subclassification of GBM. Second, it illustrates the broader concept of design and implementation of a logic model for error analysis that can be applied, with some individual modifications, to understand sources of noise and error in similar, translational investigations.

### Logical Analysis of Sources of Noise and Classification Error in the TCGA GBM Data Set

In the following section we describe the five questions used to frame our logic model and the results of the 11 individual analyses performed within the context of the model to investigate the potential sources of noise and error that result in classification inaccuracy or variability in the TCGA GBM data set.

- **Preconditions.** In order for the TCGA dataset to be appropriate for the task of molecular subclassification of GBMs into genomic subgroups with phenotypic significance, two preconditions must be met. First, there must be multiple, discrete phenotypes of GBM. This precondition is satisfied by a wealth of clinical experience demonstrating that patients with GBM have variable time to progression and recurrence, survival rates, and response to therapy [1], [2], [5], [8]. Next, genomic profiles must be useful for predicting tumor phenotype. This has been demonstrated in multiple investigations where different types of malignancies as well as different classes of the same general malignancy have been assigned to the appropriate category based upon genomic profile, including successful subclassification of GBM [8], [11], [12], [13], [14], [60], [61]. Additionally, our *multiple tumor stratification analysis (#1)* demonstrated that the TCGA GBM expression data could be used to successfully separate GBM from other malignancies, validating this specific precondition for this particular dataset.
- **Question 1: Do unbiased class discovery methods accurately identify clinical phenotypes of GBM based upon analysis of the TCGA GBM expression data?** The *unsupervised classification and clinical parameters* analysis *(#2)* addressed this question by applying several unsupervised classification algorithms to the TCGA GBM expression data. No algorithm was able to accurately stratify the tumors by subclass in any of 8 clinically-relevant, phenotypic categories. These results demonstrate that gene expression-based analysis fails to classify patients into appropriate clinical subclasses, suggesting that some type(s) of noise is present within the data and confound attempts at molecular classification. This observation differs from some of those previously reported independently by several groups, who found that phenotypically-relevant (survival) GBM subgroups could be identified based upon gene expression data [8], [12], [13], [15]. However, these previous studies were generally conducted within a single center and were performed using smaller and more homogeneous experimental populations. Tissue for these studies may have been more carefully selected, and clinical characteristics of patients whose tissues were analyzed may have been more rigorously reviewed to verify satisfaction of multiple inclusion criteria. All of these factors reduce analytic noise by limiting some of the variability within the dataset, and it is not surprising that accurate phenotypic classification is more challenging in the more heterogeneous, TCGA dataset.
- **Question 2: Do clinical and phenotypic differences exist within the data set?** One potential reason for failure of the molecular classification strategies that must be addressed at the outset would be the absence of genomic or phenotypic differences within the TCGA GBM dataset. Phenotypic variability among GBMs has been well described in general (see *preconditions*) [1], [2], [5], [8], and review of the clinical data associated with this particular dataset revealed the expected variability among each of the 8 categories analyzed in analysis *#2* (data not shown), indicating that various clinical phenotypes are also present within the dataset. Genomic variability is also well described among GBMs [8], [11], [12], [13], [14], [60], [61], and biological analysis of the unique expression signature of the outlier group identified in the *biologic analysis of the outlier group (#3)* exercise demonstrates plausible biological variability among samples in this specific data set. Accordingly, we conclude that genomic and phenotypic differences do exist in the TCGA GBM dataset.
- **Question 3: Why are underlying differences among the expression data not identified by the unsupervised analysis?** Several unsupervised, linear classifiers and dimensional reduction strategies failed to classify the TCGA GBM data into phenotypically-significant groups. The *eigenvalue data* analysis *(#4)* suggests that these classifiers (for which we use PCA as a prototype) fail to capture a large percentage (>70%) of the variability inherent in the dataset. In general, the types of linear algorithms used commonly in molecular GBM subclassification (and therefore used in this exercise) are unsuccessful when one of two, necessary criteria are not satisfied; either (1) the mean and standard deviation do not accurately and sufficiently represent the dataset, or (2) the relationships between genomic profile and clinically-relevant phenotype are either non-linear or non-orthogonal. Individually, these criteria form the basis of questions four and five.
- **Question 4: Do the mean and standard deviation accurately and sufficiently represent the dataset?** In this analysis, the mean and standard deviation may fail to appropriately represent the dataset in one of two circumstances. First, they insufficiently describe the dataset when the values of the variables serving as the basis of classification do not follow a Gaussian distribution [36]. The *expression data distribution analysis (#5)* demonstrates that the underlying data distribution in the TCGA GBM data is not purely Gaussian, with statistically-significant skew and kurtosis apparent in both the composite expression matrix and among the expression levels of the individual variables (genes). The ultimate significance of this with regard to classification requires further, dedicated investigation [36], [38], but it provides one possible explanation for the failure of linear classifiers applied to this dataset.
  A second circumstance where mean and standard deviation fail to appropriately represent the dataset is when features with the largest variance are not the primary contributors to the clinically-relevant phenotypes. Stated differently, linear classifiers fail when the signal-to-noise ratio of the dataset is low. In this context, the term *noise* is defined as the presence of measured variable values (i.e. gene expression values) that do not contribute to the intended molecular classification, and, as discussed earlier, is relative to the clinical question(s) being asked. In molecular classification experiments using gene expression data, both technical and biological noise can impede accurate classification.
  1. *Technical noise* is present when procedural artifacts are reflected in the values of the variables. We investigated potential sources of technical noise, including those associated with sample preparation *(#7)*, the array platform *(#7)*, the inclusion of low-variance or incomplete expression data *(#8)*, batch effects *(#9)*, and sample size *(#10)*. We demonstrated the presence of technical noise associated with all of these domains within the TCGA GBM dataset, although no single domain appeared to be the primary contributor to inaccurate phenotypic subclassification. Investigation of the relative magnitude of the individual contributions to the overall error and the aggregate effects of these sources of technical noise on the overall classification accuracy require detailed modeling strategies that are only beginning to be developed and that are beyond the scope of this analysis. Notwithstanding, our analysis demonstrates the importance of ongoing research into such modeling and highlights technical noise as one, important area for additional investigation.
  2. *Biological noise* [56], [73] is present when the values of the variables (gene expression values) accurately reflect underlying biology but still confound subclassification into clinically-significant subgroups. This can occur in one of three ways: (1) the expression values reflect underlying biology that does not contribute to clinically-relevant phenotypes, (2) the expression values reflect biological differences that make proportionally low magnitude contributions to clinically-relevant phenotypes, or (3) the expression values reflect biology that does contribute to clinically-relevant phenotypes, but these phenotypes either are not included in or are not detected by our analytic model. Specific analysis of the relative magnitude of the effects from each of these categories requires not only complex modeling that is beyond the scope of our investigation, but also some *a priori* knowledge of the underlying systems biology of the tumor that may not be known. These, too, must be areas of active research if the sources of error in large-scale, molecular datasets are to be accurately described.
- **Question 5: Are the relationships between features (genes) and clinically-relevant phenotype linear and orthogonal?** Linear classifiers and dimensional reduction algorithms, by definition, are designed to classify based upon linear combinations of feature values associated with the members of the dataset [36]. Additionally, many common data reduction algorithms (including PCA) require orthogonal relationships between features (or their mathematical aggregates) so that linear algebra strategies can be used to facilitate data reduction [21]. Without a detailed understanding of the systems biology of the tumor being studied, it is impossible to know for certain whether any or all relevant features are related in a linear (or orthogonal) fashion. A basic understanding of the complex principles underlying gene regulation, signal transduction, and malignant transformation, however, provide no particular basis for the assumptions of linearity or orthogonality and, in fact, may logically favor a non-linear and non-orthogonal relationship among genes. These relationships may be present across the entire expression spectrum of a given gene or above or below a specific expression threshold. Although nonlinear relationships are difficult to demonstrate definitively in incompletely-characterized biological systems, we exploited knowledge of the mathematics underlying principal component analysis and the expected relationships between the identified PCs [21], [58], [59] to provide qualitative evidence suggesting underlying non-linear and/or non-orthogonal relationships among features *(#11)*. These findings argue in favor of application of classifiers that do not assume linearity or orthogonality (e.g., kernel PCA), strategies which are now being described by array statisticians [74] but have yet to see broad application by the translational research community. Including nonlinear models in future analyses of large-volume, molecular datasets (including the TCGA data) may be a critical step to improving the accuracy of class discovery and prospective, molecular classification.

### Limitations

The investigation that we have performed focuses on the TCGA gene expression data and is therefore subject to two, general limitations inherent to this dataset. First, the TCGA expression data that we have analyzed reflects only genome-level changes in tumor biology. While gene expression is believed to correlate with phenotype, protein-level data may also be a valuable resource for molecular classification of glioblastoma [75]. Second, the TCGA gene expression data has been collected from surgical tumor specimens that necessarily contain a mixture of neoplastic and non-neoplastic cells. While this may be more significant for investigations of tumor biology than for those focusing on molecular tumor classification [8], [55], this consideration must be appreciated by investigators working with this dataset.

We also recognize that the logic model that we have described and the analysis of the TCGA GBM data that we have performed have some limitations. First, we recognize that our model may not encompass every potential source for noise or error in this particular dataset and that generalization of our model to other datasets and/or to investigations of alternate hypotheses may require inclusion or exclusion of additional confounding factors. Nonetheless, we believe that our model provides a logical framework for analyzing error that is readily expandable should modification of the specific sources of noise or error become necessary or should alternate, potential sources of error be identified. Similarly, while our logic model may be useful for prospectively exploring and limiting potential sources of error when designing novel studies, we would not support the conclusion that such a strategy necessarily assures generation of “high-quality” data and caution that such a conclusion exemplifies the logical burden-of-proof fallacy. Finally, while our model has allowed us to identify several targeted areas for future efforts to improve the quality of the TCGA GBM data, we recognize that research in additional domains may also be necessary to improve the translational potential of this dataset.

### Summary and Conclusions

We have constructed a logic model that facilitates a comprehensive analysis of potential sources of noise and error that may be responsible for some portion of the inconsistencies and lack of reproducibility observed in recent attempts to use constituent data from the TCGA GBM expression dataset to classify GBMs into clinically-relevant phenotypes. We believe that this model is a useful tool to conduct such analyses and can be readily adapted as new information regarding error and noise becomes available, and we believe it may be readily generalized to the systematic study of error in other translational domains.

Based upon our application of this logic model to the TCGA GBM expression data, we make seven conclusions regarding the potential sources of inconsistencies and error among molecular classifiers based upon the expression data comprising this dataset.

1. Because there is evidence that supports the existence of genomic and phenotypic variability within the dataset, the failure to discover genomic associations correlated with clinically-relevant phenotypes may reflect a classification failure by the common, linear classification algorithms commonly employed in this and in most other analyses of gene expression data.
2. The mean and standard deviation do not accurately and sufficiently describe either the overall expression matrix or the expression values of the individual genes contained therein.
3. Neither the distribution of the overall expression matrix nor the expression distributions of the genes or samples conform to a pure Gaussian distribution.
4. Technical noise, and likely biological noise as well, limit the signal-to-noise ratio of this dataset with respect to molecular tumor classification into phenotypically-relevant subsets.
5. Technical noise is present within the data, but none of the measured sources appear to individually represent the primary source of classification-limiting noise.
6. Biological noise is assumed to be present based upon the logic model but cannot be accurately measured using current error modeling strategies.
7. There is a suggestion of non-linear and/or non-orthogonal relationships among genes affecting clinically-relevant GBM phenotypes in this dataset.

Finally, the results of our analysis suggest that targeted research in four, specific areas may further efforts to improve classification accuracy and reproducibility by reducing noise in the TCGA dataset. These areas include: (1) additional research regarding the underlying distribution of the dataset and the implications of this distribution for classification algorithms, (2) expanded development and application of non-linear and/or non-orthogonal classifiers to this data, (3) efforts to improve modeling of technical noise, (4) and expanded efforts to model biological noise in the context of improved understanding of the systems biology of GBMs.

## Methods

### Logic Model

A logic model for directing data analysis was constructed and is depicted in Figure 1. This model was used to identify and to place in context five, fundamental questions that served as the guide for data analysis (see discussion). Based on logic model structure, eleven mathematical analyses (indicated numerically on Figure 1 and described below) were then designed to answer these five questions. Publically-available GBM gene expression data, with corresponding clinical and technical data, was prepared as described and was then used as the data source for these analyses.

### Gene Expression Datasets

The primary GBM gene expression dataset for this project was compiled from publically-available, TCGA GBM data [16]. Affymetrix® .cel files for all 340 GBM samples profiled using the Affymetrix® HT-HG-U133A chip available through TCGA as of 1 October 2009 were downloaded and were included in subsequent analyses. To facilitate biological comparisons from the analyses performed using this data set, four .cel files from normal brain tissue profiled on the same Affymetrix® platform were downloaded from GEO [63], [64], [65]. A second dataset was then constructed, including all 340 GBMs plus the four normal brain samples, which served as controls in analyses involving interpretation of tumor biology. Finally, a multiple-tumor dataset was compiled for use in the multiple tumor classification investigation. This dataset comprised all 340 GBMs plus an additional twenty (20) renal cell carcinomas (RCC) and four (4) hepatocellular carcinomas (HCC). The latter 24 samples were also profiled using the Affymetrix® HT-HG-U133A chip, and the.cel files were downloaded from GEO.

### Expression Data Preparation

Three datasets were prepared and were used in all subsequent analyses. The first was composed of all 340 GBM samples, the second comprised all 340 GBM samples plus the four normal brain controls, and the third consisted of all 340 GBM samples plus the 24 non-GBM tumors. Each dataset was normalized using the Robust Multichip Average (RMA) [22], [23], [76] with background adjustment, quantile normalization, and PLM summarization. When indicated, an alternate normalization strategy using the Affymetrix® MAS5 method [54] was utilized. All normalized expression values were log_2_ converted.

For the dataset containing normal brain tissue, and average expression value for each gene across the four control samples was computed. The expression variance among each gene was computed for the controls, and the most variable 10%, representing the genes with the least consistent expression in the control dataset, were removed from downstream analyses (as previously described). The log_2_ (tumor/normal) expression value for each of the remaining genes was computed in each of the 340 samples, and this dataset was used in subsequent analyses involving biological interpretation of data. This method of data preparation has been previously described in GBM expression analyses.

A one-sided Significance Analysis for Microarray (SAM) [27], [28] algorithm with a false discovery rate (FDR) set to zero was applied to each of the three datasets to exclude genes whose expression values were not statistically significant from baseline (zero or control). These genes do not meaningfully contribute to classification and can impede downstream analyses. These three datasets were used, as indicated, in the eleven analyses performed in this investigation.

### Clinical and Technical Data

Access to restricted clinical data was granted through the standard TCGA data access protocol. Clinical data (when available), including survival, time to progression, time to recurrence, Karnofsky performance status (KPS), sex, adjuvant chemotherapy, adjuvant radiotherapy, and presence of necrosis and endothelial proliferation in histologic sections, was merged with expression data for analyses involving these parameters. Similarly, data regarding the technical processing and specimen quality, including prior treatment, relative abundance of tumor cells, tissue block size, hospital of origin, TCGA batch number, extracted RNA concentration, OD260/280, 28S/18S, and RNA integrity number (RIN), was similarly collected and merged for analyses involving these parameters.

### Data Analysis

Microsoft Excel was used for data preparation. The TM4 software package [77], [78] was used to analyze gene expression data. Analyses performed using TM4 include hierarchical clustering and support (HCS, bootstrapping with 20 iterations) [19], k-means clustering and support [25] (KMS, 10 iterations with 80% clustering threshold), k-nearest neighbor classification (KNNC) [26], and principal component analysis [20], [21] (PCA). Differences were considered statistically-significant at *p*<0.05, unless otherwise indicated.

### PCA as a Prototypical Linear Classifier

Initial analyses used multiple classification algorithms, including HCS, KMS, and PCA to verify that all of these linear methods resulted in qualitatively-equivalent results in class discrimination investigations. Subsequently, PCA was used as a prototypical discriminator because of the robust nature of the algorithm and the logical, graphical representation of its class discrimination.

## Supporting Information

Figure S1 **Logic Model for Analyzing Variability in the TCGA GBM Dataset, Unannotated.** Logic model applied for data analysis, without annotations (see Figure 1 for annotated version and for detailed explanation). Supplied for reference purposes.
(TIF)

Table S1 **Genes Differentially Expressed between the Outlier Cluster and the Remaining GBMs.** Relative fold change values computed using 2-sided significance analysis for microarrays (SAM) with false discovery rate (FDR) = 0. Statistical significance of categorical annotations computed using the EASE score (adjusted Fisher Exact Test). 1A: Overexpressed in the outlier group relative to the remainder. 1B: Underexpressed in the outlier group relative to the remainder. 1C: EASE analysis of overrepresented categories in the overexpressed genes. 1D: EASE analysis of overrepresented categories in the overexpressed genes. 1E: Overlap between differentially expressed genes and categories in the outlier group and those identified in a previously-described GBM expression fingerprint with putative relationship to the survival phenotype [8].
(XLS)
