# Supplementary figures and images for: Why Is There a Lack of Consensus on Molecular Subgroups of Glioblastoma? Understanding the Nature of Biological and Statistical Variability in Glioblastoma Expression Data
Source: PLoS One. 2011 Jul 28;6(7):e20826. doi: 10.1371/journal.pone.0020826 (PMC3145641; doi:10.1371/journal.pone.0020826)

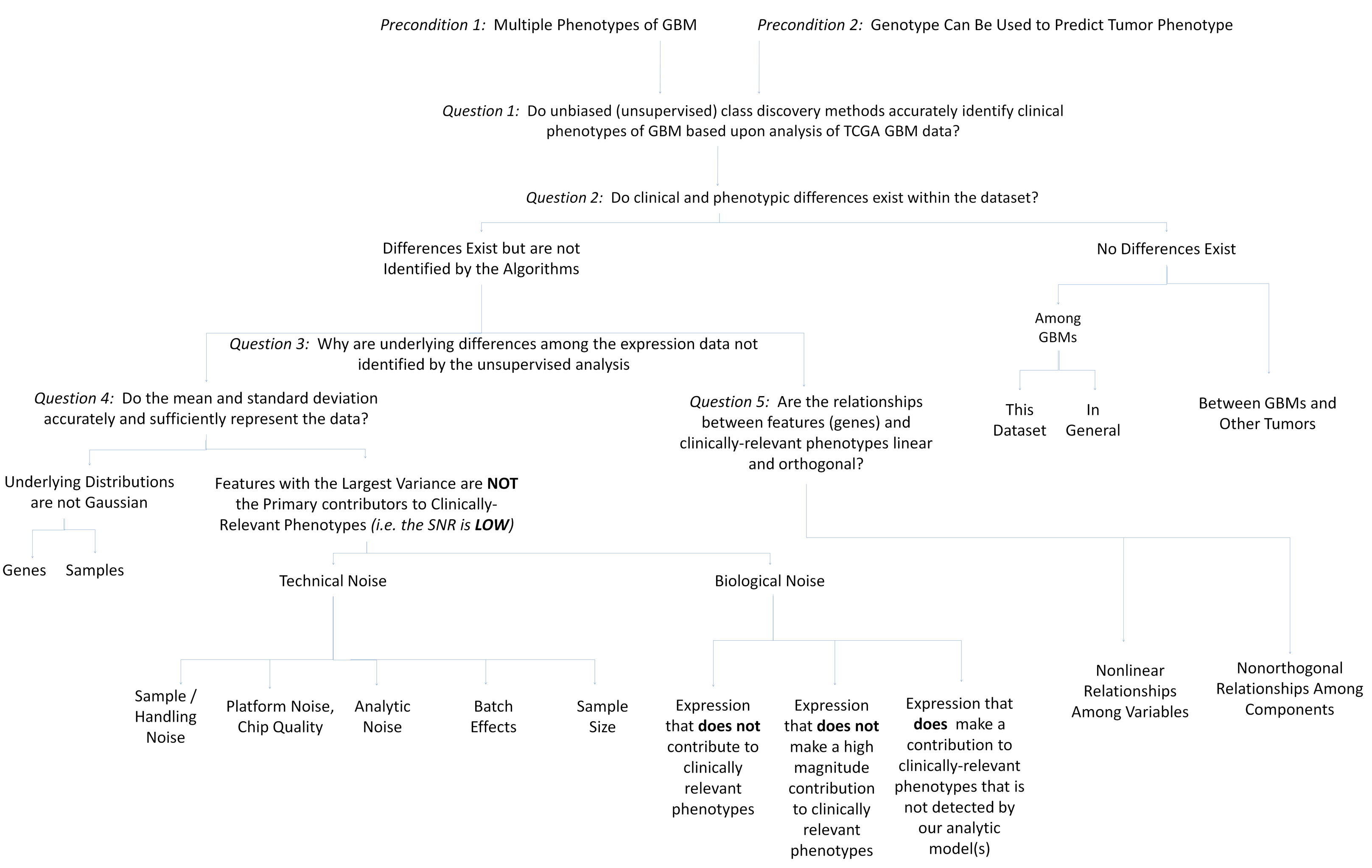

Supplement: Figure S1 — Logic Model for Analyzing Variability in the TCGA GBM Dataset, Unannotated. Logic model applied for data analysis, without annotations (see Figure 1 for annotated version and for detailed explanation). Supplied for reference purposes. (TIF) [file pone.0020826.s001.tif]
